# SARS-CoV-2 variant spike and accessory gene mutations alter pathogenesis

**DOI:** 10.1073/pnas.2204717119

**Published:** 2022-08-30

**Authors:** Marisa E. McGrath, Yong Xue, Carly Dillen, Lauren Oldfield, N. Assad-Garcia, Jayshree Zaveri, Natasha Singh, Lauren Baracco, Louis J. Taylor, Sanjay Vashee, Matthew B. Frieman

**Affiliations:** ^a^Department of Microbiology and Immunology, University of Maryland School of Medicine, Baltimore, MD 21201;; ^b^Department of Synthetic Biology and Bioenergy, J. Craig Venter Institute, Rockville, MD 20850;; ^c^Center for Pathogen Research, University of Maryland School of Medicine, Baltimore, MD 21201

**Keywords:** coronavirus, SARS-CoV-2, pathogenesis, mouse, variant

## Abstract

The ongoing COVID-19 pandemic is a major public health crisis. Despite the development and deployment of vaccines against severe acute respiratory syndrome coronavirus 2 (SARS-CoV-2), the pandemic persists. The continued spread of the virus is largely driven by the emergence of viral variants, which can evade the current vaccines through mutations in the spike protein. Although these differences in spike are important in terms of transmission and vaccine responses, these variants possess mutations in the other parts of their genome that may also affect pathogenesis. Of particular interest to us are the mutations present in the accessory genes, which have been shown to contribute to pathogenesis in the host through interference with innate immune signaling, among other effects on host machinery. To examine the effects of accessory protein mutations and other nonspike mutations on SARS-CoV-2 pathogenesis, we synthesized both viruses possessing deletions in the accessory genes as well as viruses where the WA-1 spike is replaced by each variant spike gene in a SARS-CoV-2/WA-1 infectious clone. We then characterized the in vitro and in vivo replication of these viruses and compared them to both WA-1 and the full variant viruses. Our work has revealed that the accessory proteins contribute to SARS-CoV-2 pathogenesis and the nonspike mutations in variants can contribute to replication of SARS-CoV-2 and pathogenesis in the host. This work suggests that while spike mutations may enhance receptor binding and entry into cells, mutations in accessory proteins may alter clinical disease presentation.

## Significance

A hallmark of the COVID-19 pandemic has been the emergence of severe acute respiratory syndrome coronavirus 2 (SARS-CoV-2) variants that both increased transmission and improved immune evasion. Each variant possesses mutations throughout its genome, but little is known about their effect on pathogenesis. Specifically, we are interested in the accessory genes of SARS-CoV-2, which have been shown to affect viral pathogenesis through interference with the host innate immune response. In this work, we identify accessory genes that are responsible for pathogenesis in vivo and investigate the effect of variant nonspike genes on replication and disease in mice. This work identifies accessory genes as key drivers of pathogenesis and highlights the effect of nonspike genes on replication and pathogenesis.

In December 2019, a cluster of viral pneumonia cases was observed in Wuhan, Hubei Province, China (1). The etiologic agent of this infection was found to be a novel coronavirus that we now call severe acute respiratory syndrome coronavirus 2 (SARS-CoV-2) (2). By early 2020, the virus was rapidly spreading, leading to infections on all seven continents and in every country around the world. There have since been over 550 million cases and six million deaths from this virus (3). Despite the rapid development and deployment of vaccines, the pandemic persists.

SARS-CoV-2 is a single-stranded positive-sense RNA virus that is 79% identical in sequence to SARS-CoV-1, the virus responsible for localized epidemic outbreaks beginning in February 2003 (4). The genome of this and other beta coronaviruses is composed of Open Reading Frames (ORFs), which are functionally divided between replicase proteins, structural proteins, and accessory proteins, the latter of which are unique to each CoV species (5, 6). From the 5′ to the 3′ end, the virus encodes the replicase (ORF1a/b) and the four ORFS for the structural proteins spike (S), envelope (E), membrane (M), and nucleocapsid (N). The replicase is responsible for encoding 16 nonstructural proteins that compose the replicative machinery of the virus. Additionally, interspersed with the structural proteins at the 3′ end of the genome are a variety of accessory ORFs. The accessory ORFs encode proteins that are not essential for viral replication in vitro but contribute to viral pathogenesis. The accessory ORFs of SARS-CoV-2 are very similar to those of SARS-CoV-1, and many of the functions of these ORFs have been inferred based on the previously identified functions of the SARS-CoV-1 accessory ORFs (5).

The functions of the accessory ORFs of SARS-CoV-2 involve modulation of several different host pathways including antagonism of the innate immune response. For example, SARS-CoV-2 ORF3b has been shown to antagonize interferon (IFN) signaling, and ORF7a has been shown to interfere with the IFN-stimulated gene (ISG) BST2 (7–9). SARS-CoV-2 ORF6 also participates in this antagonism of the innate immune response, as it has been shown to antagonize the IFN-induced nuclear translocation of STAT1, resulting in the reduced expression of ISGs (10). While ORF3a, ORF6, and ORF7a have been shown to be antagonists of the innate immune system, SARS-CoV-2 ORF8 has been shown to act as an agonist for the interleukin 17 (IL-17) receptor, functionally stimulating receptor signaling (11).

The continuation of the COVID-19 pandemic is largely due to the emergence of mutated strains, or “variants,” of SARS-CoV-2. The variants differ most notably in the sequence of their spike proteins, which bind to the receptor angiotensin-converting enzyme 2 (ACE2) to allow for internalization of the virus. As the spike protein is the immunodominant antigen, the emergence of variants has raised concerns regarding the breadth of protection of the SARS-CoV-2 vaccines. However, it is important to note that many of the variants of SARS-CoV-2 possess mutations in one or more of the accessory proteins. The impact of such mutations outside of the spike protein on the pathogenesis of these variants remains understudied.

To elucidate the role of the accessory proteins of SARS-CoV-2 in pathogenesis, we developed a synthetic genomics assembly approach based on transformation-associated recombination (TAR) in yeast for the creation of infectious clones of SARS-CoV-2 (12–15). We then synthesized deletion viruses of ORFs 3a/3b, 6, 7a/7b, and 8 in the prototype SARS-CoV-2 (isolate USA/-WA1/2020 or WA-1) strain of SARS-CoV-2. We then investigated the replicative fitness of these viruses in vitro before proceeding to characterization of the effect of these accessory deletions on pathogenesis in a murine model. To begin to characterize the impact of naturally occurring accessory mutations and nonspike mutations found in the variants on the pathogenesis of SARS-CoV-2, we developed recombinant variant spike proteins in the WA-1 backbone (B.1.1.7, B.1.351, and P.1). We then compared the replicative fitness in vitro of these recombinant viruses to the parent variants and characterized differences in pathogenesis in a mouse model.

## Methods

### Infectious Clone Construction and Rescue.

#### TAR-cloning SARS-CoV-2 DNA fragments.

To clone each SARS-CoV-2 DNA fragment, each of the target PCR amplicons of the genome was cloned into a TAR vector backbone for replication in budding yeast and *Escherichia coli.* TAR vectors consist of the vector backbone flanked by restriction sites to release the genomic region and homologous “hooks” of sequence. To assemble DNA fragment clones, the TAR vectors were PCR amplified from pCC1BAC-his3 with KOD Xtreme Hot Start DNA polymerase (Millipore) using the construction primers (labeled “Con”; *SI Appendix*, Table S1) (16). Then, 40 bp of homology and an I-SceI restriction site were introduced by the Con primers to each end of the vector backbone. Each DNA fragment was amplified with KOD Xtreme Hot Start DNA polymerase (Millipore) using complementary DNA (cDNA) from SARS-CoV-2/human/USA/WA-CDC-WA1/2020 and contained 80 bp of homology to its adjacent fragments for assembly. PCR products were digested with DpnI (New England Biolabs) before transformation per the manufacturer’s directions.

The SARS-CoV-2 WA-1 genome was cloned as seven individual DNA fragments: 1a-1, 1a-2, 1a-3, 1b-1, 1b-2, S, and AP. Fifty femtomoles of each WA-1 PCR amplicon was transformed with 15 fmol of the appropriate amplified YCpBAC vector into *Saccharomyces cerevisiae* strain VL6-48N spheroplasts (*MATα, his3-Δ200, trp1-Δ1, ura3-Δ1, lys2, ade2–101, met14, cir°*) as described previously (14–16). Transformants were patched on synthetic dropout medium plates without histidine. After sufficient growth, yeast cells were lysed by incubation in 25 mM NaOH at 95 °C for 30 min. Detection PCR amplification, with Q5 polymerase (New England Biolabs), lysed cell supernatant as template, and detection primers (RCO493 + “Det-F” or RCO495 + “Det-R” primers; *SI Appendix*, Table S2) were used to confirm correct junctions between the fragment and vector. Sanger sequencing of PCR products (GeneWiz, Inc.) across the SARS-CoV-2 regions was used to verify the DNA fragment clone. DNA of sequenced-validated clones was extracted from yeast patches and electroporated into *E. coli* DH10B (Thermo Fisher Scientific) (14). *E. coli* transformants were screened by colony PCR amplification for correct junction sizes with the appropriate “F” and “R” detection primers (*SI Appendix*, Table S2). Plasmid DNAs from the DNA fragment clones were isolated from *E. coli* by the Purelink HiPure Plasmid Midiprep Kit (Thermo Fisher Scientific) and modified or used for full-length genome assembly.

#### DNA fragment modifications.

In vitro CRISPR-Cas9 digestion and TAR assembly were used to make accessory ORF deletions in the AP DNA fragment. Cas9 target sites near the desired mutant coordinate were identified using Benchling’s CRISPR guide RNA design tool (https://www.benchling.com/crispr/). Single-guide RNA was transcribed from a PCR amplicon, generated as described previously, containing 18 bp of specific target sequence upstream of the PAM sequence (14). The AP DNA fragment was digested in vitro using Cas9 nuclease, *Streptococcus pyogenes* (New England Biolabs), as described previously (14). An oligonucleotide containing 40 bases of homology to either side of the target accessory ORF was TAR-assembled with each appropriate Cas9-digested AP DNA fragment in yeast by spheroplast transformation. Yeast transformants were screened for the desired mutation by Sanger sequencing of PCR amplicons (GeneWiz, Inc.) using lysed yeast culture supernatant as a template. DNA was then extracted from yeast, transformed into *E. coli*, screened for correctness, and isolated from *E. coli* as with the DNA fragment clones described above.

SARS-CoV-2 variant spike genes were generated as described previously (14). Briefly, to generate SARS-CoV-2 variant spike genes, short fragments were amplified with Platinum SuperFi II DNA polymerase (Thermo Fisher Scientific) using the wild-type S DNA fragment clone as template. Variant mutations (*SI Appendix*, Table S6) were introduced by primers into each amplicon, which had 30- to 35-bp homology at each end between the adjacent fragments. Amplicons were digested with DpnI (New England Biolabs) to remove template DNA and purified with a Qiagen PCR purification kit. Fifty femtomoles of each amplicon and 15 fmol of YCpBAC vector were assembled using a standard Gibson assembly reaction (New England Biolabs), transformed into *E. coli* DH10B competent cells (Thermo Fisher Scientific), and plated on LB medium with 12.5 mg/mL chloramphenicol. Assembled plasmids were confirmed to have the desired mutations by colony PCR amplification and Sanger sequencing (GeneWiz, Inc.) and then isolated from *E. coli* using the Purelink HiPure Plasmid Midiprep Kit (Thermo Fisher Scientific). *SI Appendix*, Table S3 lists the primers used for the construction of the variant S DNA fragments.

#### Full-length genome assembly.

The TAR vector for assembly of the full-length genome was amplified from pCC1BAC-ura3 using primers ConCMVpR and ConBGHtermF with KOD Xtreme Hot Start DNA polymerase (Millipore) (16). The cytomegalovirus (CMV) promoter was amplified from HCMV Toledo genomic DNA using primers CMVpromF and CMVpromR. Hepatitis delta virus ribozyme with bovine growth hormone (BGH) terminator fragment was obtained as a gBlock (IDT), Hu1-34. A fragment to introduce a BamHI restriction site and a fragment with a polyA region were synthesized by IDT (Pcmv-BamHI fix and PA35-HDV fix; *SI Appendix*, Table S4). Each fragment has 40 bp of homology at each end to its adjacent fragments. To generate the TAR vector for the complete genome, the pCC1-ura3 amplicon, the CMV amplicon, the BamHI fragment, the polyA fragment, and the HDVR+BGH region were assembled into circular DNA in yeast by TAR.

SARS-CoV-2 DNA fragments isolated from *E. coli* were digested with I-SceI (New England Biolabs) to release the overlapping DNA fragments from the vector, whereas the full-length TAR vector was linearized by BamHI (New England Biolabs) digestion. Fifty femtomoles of each fragment and 15 fmol of full-length genome TAR vector were mixed with yeast spheroplasts for TAR-assembly (13, 14). Transformants were patched on synthetic defined (SD) yeast medium (without Uracil) (SD-URA) plates, and positive clones were screened by PCR amplification with the detection primers listed in *SI Appendix*, Table S5. The subsequent DNA isolation from yeast and transformation into and isolation from *E. coli* were performed as described for the SARS-CoV-2 DNA fragment clones.

#### Virus reconstitution.

Twenty-four hours prior to transfection, 5e4 VeroE6 cells (American Type Culture Collection) were plated per well in 1 mL VeroE6 media (Dulbecco’s modified Eagle’s medium [DMEM]; Quality Biological), 10% fetal bovine serum (FBS; Gibco), 1% penicillin-streptomycin (Gemini Bio Products), and 1% L-Glutamine (Gibco). For transfection, 5 μg of the infectious clone and 100 ng of a SARS-CoV-2 WA-1 nucleoprotein expression plasmid were diluted in 100 μL OptiMEM (Gibco); 3 μL of TransIT-2020 (Mirus Bio) was added, and the reactions were incubated for 30 min prior to addition to the cells in the BSL-3. Cells were checked for cytopathic effect (CPE) 72 to 96 h after transfection, and the supernatant was collected for plaque purification. For plaque purification, 6e5 VeroE6 cells were plated in a 6-well plates in 2 mL VeroE6 media 24 h prior to infection; 25 μL of this supernatant was then serial diluted 1:10 in DMEM, and 200 μL of this supernatant was added to the VeroE6 cells. The cells were rocked every 15 min for 1 h at 37 °C prior to overlay with 2 mL of a solid agarose overlay (EMEM; Quality Biological), 10% FBS, 1% penicillin-streptomycin, 1% L-Glutamine, and 0.4% wt/vol SeaKem agarose (Lonza Biosciences). Cells were incubated for 72 h at 37 °C and 5% CO_2_, and individual plaques were picked and then transferred to a well of a 6-well plate with 4e5 VeroE6 cells in 3 mL VeroE6 media. After 48 h, successful plaque picks were assessed by presence of CPE; 1 mL of a well showing CPE was transferred to a T175 with 8e6 VeroE6 cells in 30 mL VeroE6 media, and the virus stock was collected 48 and 72 h after. The stocks were then titered by plaque assay.

### Titering of Virus Stocks, Growth Curve Samples, and Tissue Homogenates by Plaque Assay.

The day prior to infection, 2e5 VeroE6 cells were seeded per well in a 12-well plate in 1 mL VeroE6 media. Tissue samples were thawed and homogenized with 1 mm beads in an Omni Bead ruptor (Omni International Inc.) and then spun down at 21,000 × *g* for 2 min. A six-point dilution curve was prepared by serial dilution of 25 μL of sample 1:10 in 225 μL DMEM; 200 μL of each dilution was then added to the cells, and the plates were rocked every 15 min for 1 h at 37 °C. After 1 h, 2 mL of a semisolid agarose overlay was added to each well (DMEM, 4%FBS, and 0.06% UltraPure agarose [Invitrogen]). After 72 h at 37 °C and 5% CO_2_, plates were fixed in 2% paraformaldehyde (PFA) for 20 min, stained with 0.5 mL 0.05% Crystal Violet and 20% EtOH, and washed 2x with H_2_O prior to counting of plaques. The titer was then calculated. For tissue homogenates, this titer was multiplied by 40 based on the average tissue sample weight being 25 mg.

### Growth Curve Infection and Sample Processing.

The day prior to infection, 1.5e5 VeroE6 cells or 1.5e5 A549-ACE2 cells (courtesy of Brad Rosenberg, Icahn School of Medicine at Mount Sinai) were seeded in a 12-well plate in 1.5 mL VeroE6 media or A549-ACE2 media (DMEM, 10% FBS, and 1% penicillin-streptomycin). The day of infection, cells were washed with 500 μL DMEM, and the volume of virus needed for a multiplicity of infection (M.O.I) of 0.01 was diluted in 100 μL DMEM and added to a well in triplicate. The plates were rocked every 15 min for 1 h at 37 °C. After 1 h, the inoculum was removed, the cells were washed with 500 μL of complete media, and then 1.5 mL of complete media was added to each well; 300 μL of this supernatant was taken as the 0-h timepoint and replaced with fresh media. Then 300 μL of supernatant was pulled and replaced with fresh media at 0-, 6-, 24-, 48-, 72-, and 96-h timepoints. This supernatant was then titered.

### Infection of BALB/c and Human ACE2 (hACE2)-k18 Mice.

All animals were cared for according to the standards set forth by the Institutional Animal Care and Use Committee at the University of Maryland, Baltimore. On day 0, 12-wk-old BALB/c (Charles River Laboratories) and 12-wk-old hACE2 transgenic K18 mice (K18-hACE2) (The Jackson Laboratory) were anesthetized interperitoneally with 50 μL ketamine (1.3 mg/mouse)/xylazine (0.38 mg/mouse). The BALB/c mice were then inoculated with 1e4 plaque-forming units (pfu) of each virus in 50 μL phosphate-buffered saline (PBS). The K18-hACE2 mice were inoculated with 1e3 pfu of each virus in 50 μL PBS. The mock infected mice received 50 μL PBS only. Mice were then weighed every day until the end of the experiment. Mice were euthanized with isoflurane on day 2 and day 4. From each mouse, the left lung was collected in PFA for histology, and the right lung was split in half, with one-half placed in PBS for titer and one-half placed in TRIzol for RNA extraction. For the K18-hACE2 mice, the brain was also collected, with half of the brain in PBS for titering and the other half in TRIzol for RNA extraction.

### RT-qPCR of Tissue Homogenates.

Samples were homogenized in an Omni Bead ruptor in TRIzol, and then the RNA was extracted from 300 μL of each sample using the Direct-zol RNA miniprep kit (Zymo Research); 2 μL of isolated RNA from each sample was then converted to cDNA using the RevertAID first-strand cDNA synthesis kit (Thermo Fisher Scientific) in a 20-μL total reaction volume. For qPCR for SARS2 Rdrp, 20-μL reactions were prepared using 2 μL cDNA, 1 μL 10 mM Rdrp Forward primer (10006860; Integrated DNA Technologies), 1 μL Rdrp Reverse primer (10006881; Integrated DNA Technologies), and 10 μL 2x SYBR Green (Thermo Fisher Scientific). The reactions were then run on a 7500 Fast Dx Real-Time PCR Instrument (4357362R; Applied Biosystems). For qPCR for murine glyceraldehyde-3-phosphate dehydrogenase (GAPDH), 20-μL reactions were prepared using 2 μL cDNA, 1 μL of a 20x murine GAPDH primer (MM.pt.39a.1; Integrated DNA Technologies), and 10 μL 2x SYBR Green. The reactions were then run on a QuantStudio 5 Real-Time PCR Instrument (A28133; Applied Biosystems).

### Hematoxylin/Eosin (H&E) Staining of Lungs and Pathological Scoring.

Lungs were scored in a blinded fashion with a 0 to 5 score given, 0 being no inflammation and 5 being the highest degree of inflammation. Interstitial inflammation and peribronchiolar inflammation were scored separately. Scores were then averaged for the overall inflammation score.

### Cytokine Arrays.

The concentration of lung RNA was quantified using a Nanodrop (NanoVue Plus, GE Healthcare), and 400 ng of RNA was converted to cDNA using the Qiagen RT (2) First Strand Kit (330404; Qiagen). The cDNA was analyzed with the Qiagen RT (2) Mouse Cytokines and Chemokines array (PAMM-150Z; Qiagen). Reactions were run on a QuantStudio 5 Real-Time PCR Instrument (A28133; Applied Biosystems). The results were analyzed with the Qiagen analysis spreadsheet provided with the kit.

### RNA Sequencing and Analysis.

Library preparation and sequencing were performed by the University of Maryland Institute of Genome Sciences (Baltimore, MD). After RNA extraction as described above, transcriptomic libraries were generated and sequenced on an Illumina NovaSEq. 6000 (S4 flow cell, 100-bp paired-end; Illumina). Raw data are available in the National Center for Biotechnology Information Sequence Read Archive under the accession No. PRJNA857920. Reads were preprocessed using Cutadapt v3.4 and then aligned to the murine genome (assembly GRCm38) using STAR v2.7.8a (17, 18). Genes with a mean count of at least 10 reads in at least one condition were subjected to differential expression analysis with DESeq2 v4.1.0 followed by pathway analysis using Ingenuity Pathway Analysis (Qiagen) (19). Genes were only considered for follow-up if the magnitude of differential expression was at least twofold in either direction and the difference in expression between conditions was significant (*P* < 0.05) after multiple testing correction.

### Statistical Analysis.

All statistical analyses were carried out using GraphPad Prism software (GraphPad Software) or R version 4.1.1 (20). The cutoff value used to determine significance was *P* ≤ 0.05 for all tests. The statistical tests run were unpaired *t* tests assuming unequal variances or one-way ANOVA followed by T-test with Bonferroni correction, where indicated. For the differential expression analysis, reported *P* values are the result of the Wald test after Benjamini-Hochberg correction, as calculated by DESeq2 v4.1.0 (19).

### Biosafety Approval.

All virus experiments and recombinant virus creation were approved by the Institutional Biosafety Committee at The University of Maryland, Baltimore. At their request, the P.1 S in WA-1 virus has been destroyed due to its increased in vivo pathogenesis compared to the clinical P.1 strain.

## Results

### Infectious Clone System.

To enable rapid and combinatorial modification of SARS-CoV-2, we established a yeast-based assembly approach using TAR to assemble a complete genome from overlapping DNA fragments. This approach is based on one that we previously used to engineer human herpesviruses (14, 15). It involves first generating a set of sequence-validated DNA fragments that encompass a complete genome, which can then be modified individually and in parallel to obtain DNA fragments containing the desired changes. The modified fragments can be mixed and matched with unmodified DNA fragments for assembly into complete genomes to generate the desired mutant viruses.

**Fig. 1.**
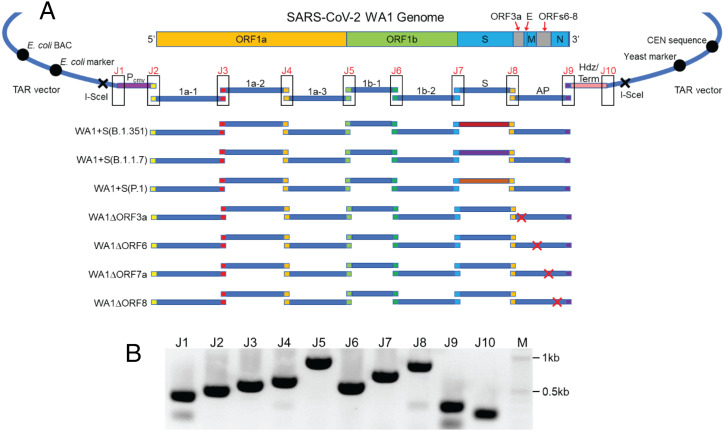
Assembly of infectious clone genomes of SARS-CoV-2. (*A*) The genome of WA1 was assembled from sequence-validated overlapping (colored ends) DNA fragments (1a-1, 1a-2, 1a-3, 1b-1, 1b-2, S, AP; blue lines) by TAR in yeast. The infectious clone genomes can be maintained in yeast and *E. coli* by a YCpBAC vector. The infectious clone genome, which is flanked by I-SceI sites, is driven by a CMV promoter (P_cmv_) and has a hepatitis delta virus ribozyme sequence (Hdz) as well as a BGH terminator (Term) at the 3′ end of the genome. SARS-CoV-2 WA1 genomes containing either spike variants or accessory ORF deletions were assembled from a mix of unmodified and appropriate modified DNA fragments. (*B*) PCR amplifications of assembly junctions (J1to J10) to confirm a full-length genome in *E. coli.* M, 2-log marker.

For SARS-CoV-2, the genome was deconstructed into seven individual DNA fragments: three for ORF1a (1a-1–1a-3), two for ORF1b (1b-1 and 1b-2), one for S, and one for the accessory protein 3′ genome region (AP) (Fig. 1*A*). Each individual fragment was TAR-cloned by transforming into yeast. Each target fragment was amplified by PCR using SARS-CoV-2 WA-1 cDNA as a template with the primer pairs listed in *SI Appendix*, Table S1 together with a PCR-amplified YCpBAC vector that contained each set of appropriate homologous sequences at either end (*SI Appendix*, Table S1). Each individual DNA fragment was confirmed by junction PCR amplification using a primer homologous to the YCpBAC vector and a primer homologous to the target DNA fragment (*SI Appendix*, Fig. S1 and Table S2). Positive yeast clones were then transformed into *E. coli* to enable isolation of large amounts of DNA and were again confirmed by junction PCR amplification (*SI Appendix*, Fig. S1). Finally, each individual DNA fragment, which was validated by Sanger sequencing, was flanked by I-SceI sites and contained 80 bp of homologous sequence to its adjacent fragments. Deletion of the accessory genes was performed at the DNA fragment level (AP) by cleaving with Cas9 protein in vitro guided by guide RNAs specific to each accessory gene and then transforming into yeast each cleaved DNA fragment together with the appropriate “fix” that contains 40 bases of homology to either side of the coding region of the target accessory gene (*SI Appendix*, Table S3). Positive clones were identified by PCR amplification across the target gene and then transformed into *E. coli* for large-quantity isolation of DNA (*SI Appendix*, Fig. S2). The Spike ORFs from select SARS-CoV-2 variants were generated by rebuilding the WA-1 S DNA fragment by Gibson assembly in *E. coli* from PCR-amplified products using primers to introduce the changes where needed and the WA-1 S DNA fragment as a template (*SI Appendix*, Table S3). Each of the SARS-CoV-2 S variants generated was validated by Sanger sequencing. Finally, to generate the different full-length genomes by TAR assembly, the appropriate sets of DNA fragments (1a-1 to AP) were cleaved with I-SceI to separate the SARS-CoV-2 fragment from the YCpBAC vector and then transformed into yeast together with a YCpBAC vector that contained a CMV promoter and homology to DNA fragment 1a-1 at one end and homology to DNA fragment AP, Hepatitis delta virus ribozyme sequence, as well as BGH terminator at the other end (Fig. 1*A* and *SI Appendix*, Table S4). Correct assembly of the full-length genomes was confirmed by junction PCR amplification (Fig. 1*B*) using primer pairs that span the SARS-CoV-2 genome (*SI Appendix*, Table S5). Positive clones were transferred to *E. coli* for isolation of SARS-CoV-2 full-length genome DNA.

Following confirmation of the successful synthesis of each full-length genome clone, VeroE6 cells were transfected with each isolated YCpBAC. At 72 to 96 h later, the cells were monitored for CPE, and supernatant was collected. The virus was then plaque purified and stocked. The accessory ORF deletion viruses of WA-1 were successfully recovered and sequence-verified prior to use in experiments.

#### Replication of SARS-CoV-2 deletion viruses in cells.

**Fig. 2.**
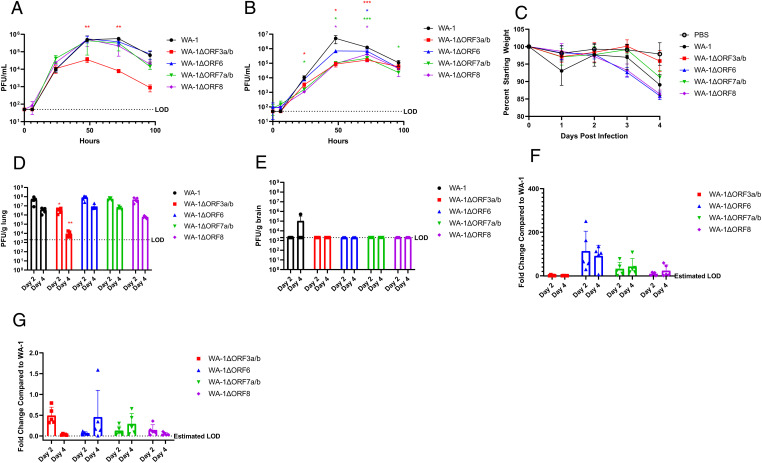
WA-1 accessory deletion viruses in 12-wk-old K18-hACE2 mice. (*A*) Supernatant titers of VeroE6 cells infected with an M.O.I. of 0.01 of accessory deletion viruses with supernatant pulled at 0, 6, 24, 48, 72, and 96 h and titered by plaque assay. (*B*) Supernatant titers of A549-ACE2 cells infected with an M.O.I. of 0.01 of accessory deletion viruses with supernatant pulled at 0, 6, 24, 48, 72, and 96 h and titered by plaque assay. (*C*) Percent starting weight on days 0 to 4 of K18-hACE2 mice infected with 1e3 pfu of each accessory deletion virus. (*D*) Lung viral titers of mice euthanized on day 2 and day 4 by pfu/g lung. (*E*) Brain viral titers of mice euthanized on day 2 and day 4 by pfu/g brain. (*F*) Lung viral loads of mice euthanized on day 2 and day 4 by qPCR for Rdrp. (*G*) Brain viral loads of mice euthanized on day 2 and day 4 by qPCR for Rdrp. LOD = Limit of Detection. *P ≤ 0.05; **P ≤ 0.005; ***P ≤ 0.0005. Error bars are standard deviation.

The growth of the wild-type SARS-CoV-2 (WA-1) and the accessory ORF deletion series was analyzed in the IFN-β–incompetent VeroE6 cells and in the IFN-β–competent A549-hACE2 cells. To evaluate the replicative kinetics of the deletion viruses in vitro, VeroE6 cells were infected with an M.O.I. of 0.01 of WA-1, WA-1ΔORF3a/b, WA-1ΔORF6, WA-1ΔORF7a/b, and WA-1ΔORF8. Supernatant was titered at 0, 6, 24, 48, 72, and 96 h after infection by plaque assay. Compared to the wild-type WA-1 virus, WA-1ΔORF3a/b was the only accessory gene deletion virus to show an attenuated phenotype in these IFN-incompetent cells, demonstrating significantly attenuated growth at 48 and 72 h (Fig. 2*A*; *P* = 0.0027 and *P* = 0.0014, respectively).

To analyze the replicative kinetics of the accessory deletion viruses in IFN-competent cells, growth curves were also performed in A549-ACE2 cells (Fig. 2*B*). Unlike in the VeroE6 cells, all of the accessory deletion viruses exhibited some attenuation in these IFN-competent cells compared to WA-1. WA-1ΔORF3a/b was significantly attenuated at 24 h (*P* = 0.025), 48 h (*P* = 0.041), and 72 h (*P* = 0.00021). WA-1ΔORF6 was attenuated only at 72 h (*P* = 0.032). WA-1ΔORF7a/b was attenuated at 24 h (*P* = 0.010), 48 h (*P* = 0.041), 72 h (*P* = 0.00040), and 96 h (*P* = 0.036). WA-1ΔORF8 was also attenuated at 48 h (*P* = 0.041) and 72 h (*P* = 0.012). The increased attenuation of these accessory deletion viruses in IFN-competent cell lines suggests a significant role of IFN antagonism in viral pathogenesis in vitro.

#### WA-1 accessory deletion viruses in K18-hACE2 mice.

We sought to identify the role of the accessory proteins in SARS-CoV-2 pathogenesis using a mouse model of COVID-19. C57BL/6 K18/hACE2 (K18-hACE2) are highly permissive to SARS-CoV-2 due to the widespread expression of hACE2. This hACE2 is expressed under the control of the keratin 18 (K18) promoter, leading to hACE2 expression in almost all cells of the mouse. Previous reports have shown this mouse strain to be permissive to wild-type SARS-CoV-2 infection without the need for any adaptations or mutations in the spike protein. We utilized these mice in the comparison of wild-type and deletion strains so that no alterations to the spike protein would be needed for replication. To evaluate the role of each accessory protein, 12-wk-old K18-hACE2 mice were infected with 1e3 pfu of each virus, weighed daily, and euthanized at 2 and 4 d after infection for collection of tissue samples. Wild-type WA-1 displayed ∼12% weight loss through 4 d of infection, with similar weight loss for WA-1ΔORF6, WA-1ΔORF7a/b, and WA-1ΔORF8 viruses (Fig. 2*C*). However, infection with WA-1ΔORF3a/b resulted in an attenuated weight loss phenotype in the mice, with these mice losing less than 4% of their starting weight (Fig. 2*C*).

Lung titers for wild-type and deletion viruses were analyzed at days 2 and 4 after infection to determine if there were differences in replicative fitness between the mutants and WA-1. Lung titers for WA-1 reached 7e7 and 8e6 pfu/g lung at days 2 and 4, respectively (Fig. 2*D*). The mutant viruses WA-1ΔORF6, WA-1ΔORF7a/b, and WA-1ΔORF8 had similar lung titers to WA-1 at day 2. This was also found with infection of WA-1ΔORF6 and WA-1ΔORF7a/b at day 4. WA-1ΔORF8 did exhibit a one-log reduction in lung titer at day 4, although this reduction was not significant (Fig. 2*D*). Consistent with the observed attenuation in weight loss, mice infected with WA-1ΔORF3a/b had significantly attenuated lung titers at both day 2 and day 4 with one log–lower lung titer at day 2 (*P* = 0.012) and three log–lower titer at day 4 after infection (*P* = 0.0030) compared to WA-1 (Fig. 2*D*).

Lung viral RNA was analyzed for WA-1 and each mutant virus at days 2 and 4 after infection. The levels for viral RNA for each of the deletion viruses were compared to the RNA levels by fold change in the lungs of the mice infected with the wild-type virus. Although none of the fold changes were significant, mice infected with the deletion viruses for ORF6, ORF7a/b, and ORF8 showed higher viral RNA levels compared to WA-1, while mice infected with the ORF3a/b deletion virus showed slightly lower lung RNA levels compared to WA-1 (Fig. 2*F*).

Due to the overexpression of hACE2 in neural tissues in the K18-hACE2 mice, SARS-CoV-2 can invade and replicate in the brain of these mice. We therefore assessed the replication of WA-1 and the accessory deletion viruses in brain tissue as well. For all of the accessory deletion viruses, there were undetectable live virus titers in the brain at day 2 and day 4 after infection (Fig. 2*E*). Despite there being little virus in the brain by plaque assay, there was viral RNA detected in the brain in levels that were not significantly different between the accessory deletions when compared to WA-1 (Fig. 2*G*).

**Fig. 3.**
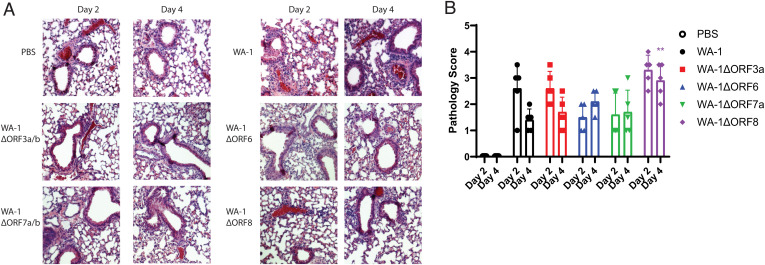
Lung pathology of 12-wk-old K18-hACE2 mice infected with accessory deletion viruses of WA-1. (*A*) H&E-stained sections of the lungs. (*B*) Pathological scoring of the lungs. *P ≤ 0.05; **P ≤ 0.005; ***P ≤ 0.0005. Error bars are standard deviation.

Lungs were fixed and stained with H&E to determine whether inflammation and lung damage were different between the wild-type WA-1 and deletion mutants. They were scored in a blinded fashion with a 0 to 5 score given, 0 being no inflammation and 5 being the highest degree of inflammation. Interstitial inflammation and peribronchiolar inflammation were scored separately, and then scores were averaged for the overall inflammation score. Despite the attenuation of weight loss with infection with WA-1ΔORF3a/b, there was no significant difference in lung pathology in the mice when compared to WA-1 (Fig. 3 *A* and *B*). Across the accessory deletion panel, there were no significant differences in lung pathology seen with any of the accessory deletion viruses except for an unexpected increase in lung pathology scores for mice infected with WA-1ΔORF8 at day 4 (*P* = 0.0037; Fig. 3 *A* and *B*). The increased pathology of WA-1ΔORF8 is intriguing because of the reported immunomodulatory role of the secreted ORF8 protein in pathogenesis. This finding will be studied in future experiments.

### Creation of Infectious Clones of Variant Spikes in a WA-1 (icWA-1) Background and Analysis in Cells.

The spike ORFs from selected SARS-CoV-2 variants were cloned into the icWA-1 clone to replace the WA-1 spike ORF. The goal of these mutant viruses was to dissociate the effects of the variant spike ORF from the other mutations in the variant genomes. This allowed for the separation of spike-specific pathogenesis differences in the variants from the differences that resulted from the nonspike mutations when we compared these spike variant viruses to both WA-1 and the full variant virus.

**Fig. 4.**
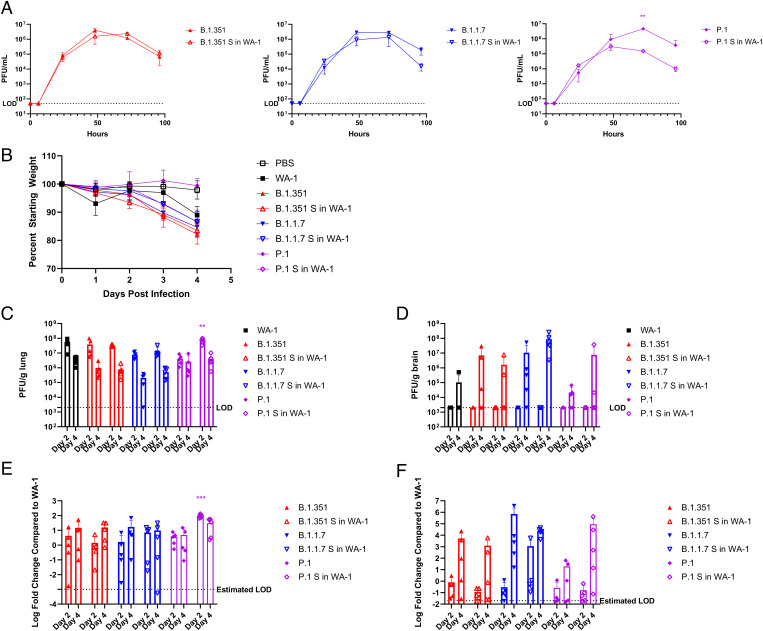
Variant spikes in WA-1 viruses in 12-wk-old K18-hACE2 mice. (*A*) Supernatant titers of VeroE6 cells infected with an M.O.I. of 0.01 of variant spikes in WA-1 and parent variant viruses, with supernatant pulled at 0, 6, 24, 48, 72, and 96 h and titered by plaque assay. (*B*) Percent starting weight on days 0 to 4 of K18-hACE2 mice infected with 1e3 pfu of each variant spike in WA-1 virus or the parent variant virus. (*C*) Lung viral titers of mice euthanized on day 2 and day 4 by pfu/g lung. (*D*) Brain viral titers of mice euthanized on day 2 and day 4 by pfu/g brain. (*E*) Lung viral loads of mice euthanized on day 2 and day 4 by qPCR for Rdrp. (*F*) Brain viral loads of mice euthanized on day 2 and day 4 by qPCR for Rdrp. LOD = Limit of Detection. *P ≤ 0.05; **P ≤ 0.005; ***P ≤ 0.0005. Error bars are standard deviation.

The spike variant viruses were cloned, rescued, sequenced, and analyzed for their replication in vitro and in vivo. Upon successful recovery and sequence verification of the variant spikes in WA-1 viruses, the replication of these viruses was compared to the parent variants at an M.O.I. of 0.01 in VeroE6 cells. The variant spike and parental variant pairs, B.1.351 and B.1.351 S in WA-1 and B.1.1.7 and B.1.1.7 S in WA-1, showed no difference in supernatant viral titers at all timepoints tested (Fig. 4*A*). The supernatant titer for P.1 S in WA-1 was significantly lower than the parent variant P.1 at only the 72-h timepoint (Fig. 4*A*; *P* = 0.0015).

### Variant Spike in WA-1 Viruses Compared to Variant Clinical Isolates in K18-hACE2 Mice.

The variant spike viruses were then tested to determine if their pathogenesis in mice was different from the parent clinical isolates that have the spike mutations and the full composition of nonspike mutations. To investigate differences in pathogenesis in vivo, 12-wk-old K18-hACE2 mice were infected with 1e3 pfu of each virus. Mice were weighed every day and were euthanized at either day 2 or day 4 after infection for collection of lung tissue and brain tissue. Reflective of the in vitro results, mice infected with B.1.351 and mice infected with B.1.351 S in WA-1 displayed similar weight loss of around 12%. Mice infected with B.1.1.7 and mice infected with B.1.1.7 S in WA-1 also exhibited similar weight loss of around 10% (Fig. 4*B*). However, mice infected with P.1 S in WA-1 showed a weight loss of 12% despite there being very minimal weight loss in mice infected with the P.1 parent variant (Fig. 4*B*). The lung titers of the mice reflected the weight loss, with mice infected with B.1.351 S in WA-1 or B.1.17 S in WA-1 displaying similar lung titers of around 10^8^ on day 2 and 10^6^ on day 4, which was seen in the mice infected with the parent variants (Fig. 4*C*). Mice infected with P.1 S in WA-1 had significantly higher lung titers at day 2 compared to mice infected with P.1, with lung titers being roughly one log higher at this time (Fig. 4*C*; *P* = 0.00057).

As with the accessory deletion viruses, viral titers in the brain were also analyzed. Although none of the infected mice had measurable titer in the brain at day 2, the brain titers for day 4 trended with the lung titer results, with B.1.351 and B.1.351 S in WA-1 and B.1.1.7 and B.1.1.7 S in WA-1 showing similar titers of ∼10^7^ to 10^8^ at day 4 (Fig. 4*D*). P.1 S in WA-1 showed two log–higher titer in the brain at day 4 compared to P.1, although this was not statistically significant (Fig. 4*D*).

Viral RNA levels were quantified in lung and brain to compare with live virus titer results. The viral RNA levels in the lung and in the brain were reflective of the titers (Fig. 4 *E* and *F*), with the only significant difference being between the day 2 lung RNA levels of P.1 and P.1 S in WA-1 (Fig. 4*E*; *P* = 0.00070).

**Fig. 5.**
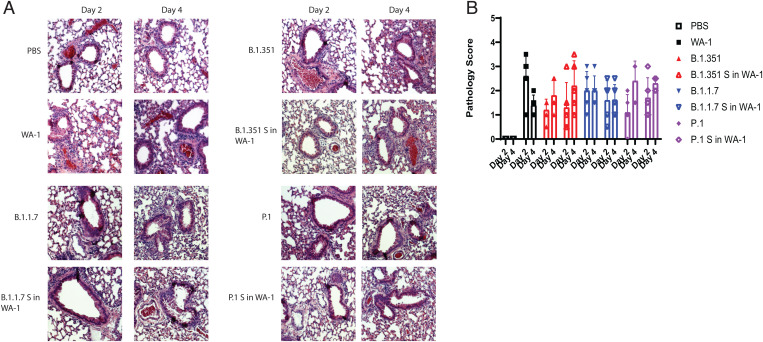
Lung pathology of 12-wk-old K18-hACE2 mice infected with variant spikes in WA-1 and parent variants. (*A*) H&E-stained lung sections. (*B*) Pathological scoring of the lungs.

Lung inflammation was also analyzed across clinical isolates and spike mutant viruses. The lungs for the variant spike in WA-1 mice looked similar in terms of inflammation to the lungs of the mice infected with the parental variant viruses (Fig. 5*A*). Although there were no significant differences in lung inflammation between the parent variant viruses and the variant spike in WA-1 viruses, the P.1 S in WA-1 lungs had higher lung pathology scores on both day 2 and day 4 compared to the P.1 mice, which trends with both the titer and the weight loss data (Fig. 5*B*).

### Variant Spikes in WA-1 in BALB/c Mice.

**Fig. 6.**
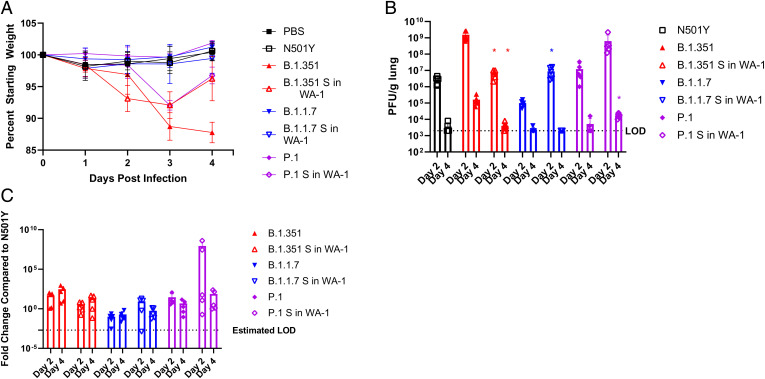
Variant spikes in WA-1 viruses in 12-wk-old BALB/c mice. (*A*) Percent starting weight on day 0 to 4 of BALB/c mice infected with 1e4 pfu of each variant spike in WA-1 virus. (*B*) Lung viral titers of mice euthanized on day 2 and day 4 by pfu/g lung. (*C*) Lung viral titers of mice euthanized on day 2 and day 4 by qPCR for Rdrp. LOD = Limit of Detection. *P ≤ 0.05; **P ≤ 0.005; ***P ≤ 0.0005. Error bars are standard deviation.

The wild-type SARS-CoV-2/WA-1 virus does not replicate in wild-type mice due to poor interactions with mouse ACE2. The N501Y mutation in spike does, however, allow for replication in wild-type mice. Interestingly, the variants examined in this study did have the N501Y mutation, allowing for comparison of pathogenesis between recombinant virus variants and their parent strains in BALB/c mice. For this comparison, 12-wk-old BALB/c mice were infected with 1e4 pfu of each virus and euthanized at either day 2 or day 4 after infection for analysis. As we observed with the K18-hACE2 mouse experiment, infection with P.1 S in WA-1 resulted in increased weight loss of around 5% compared to infection with P.1, which produced no weight loss. Both B.1.1.7 and B.1.1.7 S in WA-1 infection produced minimal weight loss in these mice. Unlike the results from the infection in K18-hACE2 mice, mice with B.1.351 S in WA-1 showed attenuated weight loss of around 7% compared to B.1.351, which produced roughly 12% weight loss (Fig. 6*A*).

The viral titers in the lung homogenates were mostly reflective of the weight loss findings, with infection with B.1.351 S in WA-1 producing significantly lower lung titers by about two logs at day 2 and day 4 compared to infection with B.1.351 (Fig. 6*B*; *P* = 0.008 and *P* = 0.014, respectively). Despite there being no differences in weight loss, B.1.1.7 S in WA-1 infection resulted in significantly higher lung titers by two logs at day 2 after infection (*P* = 0.013) compared to B.1.1.7, with both viruses being nearly cleared by day 4 after infection (Fig. 6*B*). Infection with P.1 S in WA-1 resulted in higher lung titers by two logs at day 2 and one log at day 4 compared to infection with P.1, although only the day 4 titers differed significantly (Fig. 6*B*; *P* = 0.011).

**Fig. 7.**
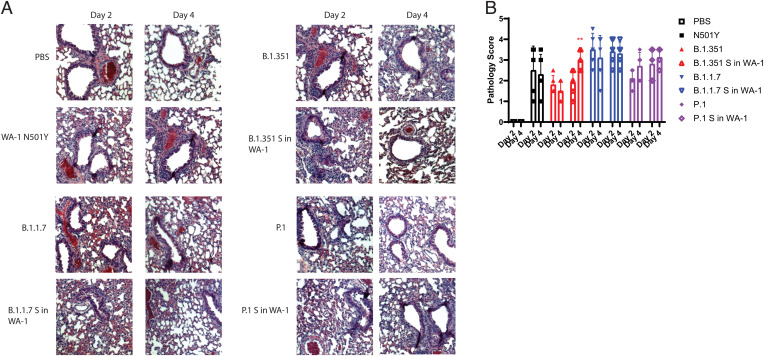
Lung pathology of 12-wk-old BALB/c mice infected with variant spike in WA-1 viruses and parent variants. (*A*) H&E-stained lung sections. (*B*) Pathological scoring of the lungs. *P ≤ 0.05; **P ≤ 0.005; ***P ≤ 0.0005. Error bars are standard deviation.

The viral RNA levels in the lungs at these timepoints correlated to the viral titer levels, although none of these findings were statistically significant (Fig. 6*C*). Lung pathology was also analyzed. The lungs of mice infected with B.1.1.7 S in WA-1 showed similar pathology to the mice infected with B.1.1.7, and this was also reflected by the pathology scores (Fig. 7 *A* and *B*). The lungs of mice infected with P.1 S in WA-1 showed increased inflammation compared to the lungs of mice infected with P.1 on both day 2 and day 4 (Fig. 7 *A* and *B*) after infection. Despite B.1.351 S in WA-1 producing attenuated weight loss, the lung pathology scores for these mice were higher compared to mice infected with B.1.351, although none of the lung pathology differences were statistically significant (Fig. 7 *A* and *B*).

### Lung Cytokine Profiling for WA-1, WA-1ΔORF3a, P.1, and P.1 S in WA-1.

We then analyzed the host response to selected viruses that showed the most significant differences in weight loss and titer compared to the parent strains. We compared the inflammatory cytokine and chemokine profile by qPCR on both the day 2 lungs and the day 4 lungs of the K18-hACE2 mice infected with WA-1, WA-1ΔORF3a/b, P.1, and P.1 S in WA-1 (*SI Appendix*, Table S7 and Dataset S1). It is interesting to note that for both the WA-1ΔORF3a/b to WA-1 comparison and the P.1 S in WA-1 to P.1 comparison, the majority of significant cytokine and chemokine changes were seen at day 2. For this reason, we will focus on the changes we saw at day 2.

For K18-hACE2 mice infected with WA-1ΔORF3a/b, the majority of significant fold changes in cytokine and chemokine levels compared to mice infected with WA-1 were less than 1. The top five significantly down-regulated genes were Ccl7, Csf3, Cxcl1, Cxcl3, and Cxcl5, which are key cytokines involved in neutrophil production and recruitment (Table 1). A small subset of genes were significantly up-regulated. These genes were Adipoq, Bmp7, Ctf1, Cxcl11, Il4, and Il5, which are mostly cytokines involved in cell survival and the early stages of T cell activation (Table 1).

**Table 1.** Fold changes of inflammatory chemokines and cytokines in day 2 lungs of mice infected with WA-1ΔORF3a/b compared to mice infected with WA-1

Experimental group	WA-1ΔORF3a/b			
Control group	WA-1			
	Significantly up-regulated genes	Fold change	Significantly down-regulated genes	Fold change
	Adipoq	9.64	Ccl1	0.45
	Bmp7	2.23	Ccl12	0.37
	Ctf1	2.38	Ccl2	0.17
	Cxcl11	2.11	Ccl20	0.31
	Il4	2.13	Ccl24	0.46
	Il5	2.23	Ccl3	0.29
			Ccl4	0.21
			**Ccl7**	**0.16**
			Cd70	0.48
			**Csf3**	**0.08**
			**Cxcl1**	**0.13**
			Cxcl10	0.49
			**Cxcl3**	**0.09**
			**Cxcl5**	**0.06**
			Cxcl9	0.36
			Ifng	0.46
			Il10	0.45
			Il11	0.48
			Il17a	0.40
			Il1a	0.48
			Il1b	0.23
			Il1rn	0.23
			Il2	0.45
			Il21	0.44
			Il22	0.44
			Il23a	0.34
			Il24	0.37
			Il3	0.44
			Il6	0.27
			Il9	0.44
			Nodal	0.44
			Osm	0.31
			Tnf	0.31

As was seen with the WA-1ΔORF3a and WA-1 comparison, the majority of significant fold changes occurred at day 2 for the P.1 and P.1 S in the WA-1 comparison. There were few differences in gene induction when comparing P.1 and P.1 S in WA-1 in relation to the WA-1ΔORF3a/b and WA-1 differentially expressed genes. The top five up-regulated genes were Ccl1, Ccl20, Csf3, Cxcl5, and Osm, which are involved in the attraction of neutrophils and lymphocytes (Table 2). All of the down-regulated genes had similar fold changes. These genes include Csf2, Il12a, and Il21, which are known to be secreted by innate immune cells, including natural killer cells (Table 2).

**Table 2.** Fold changes of inflammatory chemokines and cytokines in day 2 lungs of mice infected with P.1 S in WA-1 compared to mice infected with P.1

Experimental group	P.1 S in WA-1			
Control group	P.1			
	Significantly up-regulated genes	Fold change	Significantly down-regulated genes	Fold change
	**Ccl1**	**2.91**	Adipoq	0.36
	Ccl2	2.66	Csf2	0.41
	**Ccl20**	**2.71**	Il12a	0.35
	Ccl7	2.47	Il21	0.46
	**Csf3**	**3.49**	Il5	0.48
	Cxcl1	2.47	Mstn	0.17
	**Cxcl5**	**4.23**	Thpo	0.46
	Il1b	2.02	Tnfsf11	0.36
	Il1rn	2.18		
	Il23a	2.65		
	Il24	2.15		
	Il6	2.47		
	**Osm**	**2.72**		

### Transcriptomic Analysis of SARS-CoV-2 Accessory Deletion Viruses in K18-hACE2 Mice.

**Fig. 8.**
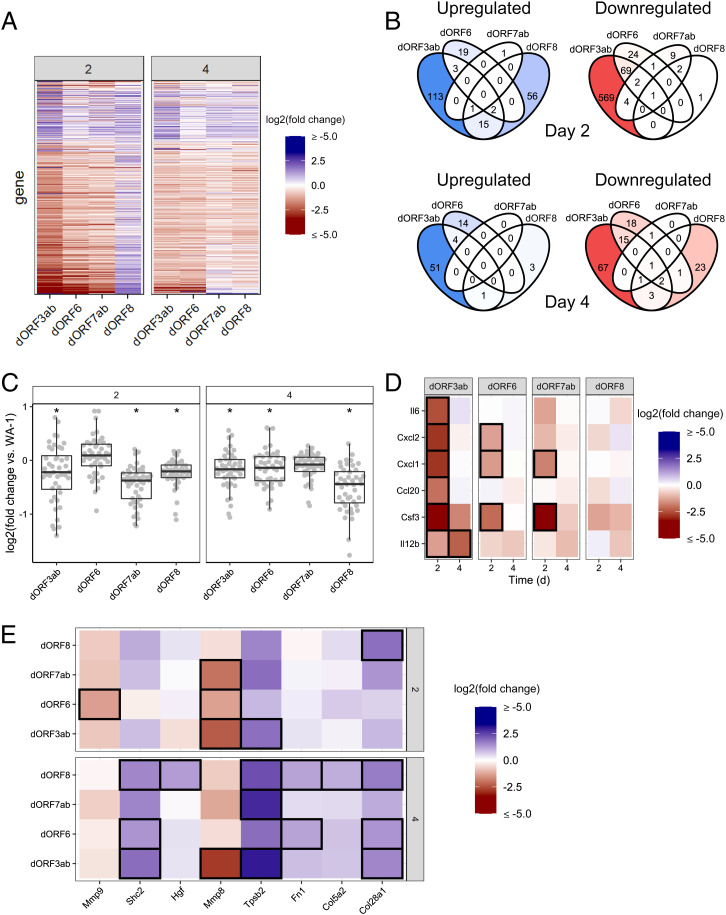
Transcriptional response of mouse lungs after infection with wild-type and accessory deletion viruses. (*A*) Overview heatmap showing all genes differentially expressed in at least one condition from murine lungs infected with accessory deletion viruses, relative to WA-1 at day 2 (*Left*) or 4 (*Right*). Blue indicates higher expression relative to WA-1; red indicates reduced relative expression. A table with all gene names and fold changes is included in *SI Appendix*, Table S8*A*; this table includes values for all other heatmaps in this figure. (*B*) Venn diagrams showing the number of genes for each variant and combinations that are significantly different from WA-1 at day 2 (*Top*) and day 4 (*Bottom*). Up-regulated genes are shown in the blue-shaded diagrams (*Left*) and down-regulated in red (*Right*). (*C*) Fold changes of murine ISGs in accessory deletion viruses relative to WA-1. A table with all gene names and fold changes is included in *SI Appendix*, Table S8*B*. Conditions with a significant change in mean ISG expression compared to wild-type infection (mean fold change not equal to 0) are indicated by an asterisk (*P* < 0.05, one-way ANOVA followed by one-way T-test with Bonferroni correction). Error bars are standard deviation. (*D*) Heatmap showing IL-17–regulated gene expression relative to WA-1. Genes that met our significance threshold (at least twofold change in expression, adjusted *P* value < 0.05) in a particular condition are outlined in black. (*E*) Expression of genes in the wound healing signaling pathway in knockout infections relative to WA-1. Genes that met our significance threshold (at least twofold change in expression, adjusted *P* value < 0.05) in a particular condition are outlined in black.

To better understand the host response to infection, we performed RNA sequencing of total RNA on the lungs of the K18-hACE2 mice infected with the accessory deletion viruses at both 2 and 4 d after infection. We found that while the ORF3ab, ORF6, and ORF7a/b deletions all had transcriptionally similar host responses across differentially expressed genes, the transcriptomic profiles of the mice infected with the ΔORF8 virus demonstrated substantially different host responses compared to WA-1 (Fig. 8). Differentially expressed genes were calculated for each virus compared to WA-1 at the same timepoint (Fig. 8*A*). We found significant down-regulation of genes in WA-1ΔORF3a/b–, WA-1ΔORF6–, and WA-1ΔORF7a/b–infected lungs compared to WA-1–infected lungs at day 2, with gene expression levels becoming more similar by day 4. WA-1ΔORF8–infected mice had noticeably different gene regulation at day 2 after infection, with a large number of genes up-regulated compared to WA-1. This pattern was reduced by day 4 after infection, where WA-1ΔORF8–infected mouse lungs looked more similar to WA-1–infected lungs (Fig. 8*A*). Venn diagrams were produced to show overlapping of specific genes across the lungs of mice infected with each virus. This showed significant differences between each deletion virus and the other viruses analyzed, with less difference found at day 4 after infection than at day 2, similar to the heat maps (Fig. 8*B*).We compared a large panel of ISGs across all infections and found that infection with both the WA-1ΔORF3a/b and WA-1ΔORF8 viruses induced lower ISG expression levels at both day 2 and day 4 than WA-1 did, with the dORF8 virus having the lowest magnitude across all conditions at day 4 (Fig. 8*C*).

Due to the published finding that ORF8 is secreted and induces the IL-17 signaling pathway, we analyzed key IL-17–induced genes to determine if WA-1ΔORF8–infected mouse lungs have reduced IL-17 pathway induction (11). We found that there was little difference between WA-1ΔORF8–infected mouse lungs and WA-1–infected mouse lungs at this dose in this model (Fig. 8*D*).

Pathway analysis was performed on these differentially expressed genes, and we found that in the WA-1ΔORF8–infected lung tissue, there was a statistically significant increase in genes associated with lung damage and wound healing, with these genes being up-regulated at day 4 after infection (Fig. 8*E*). The histological examination of WA-1ΔORF8 lungs showed that there was increased inflammation and damage in lungs at day 2 and day 4 after infection (Fig. 3). This suggests that the WA-1ΔORF8 virus causes more lung damage and inflammation, which may be due to its lack of IL-17 pathway induction. Together, these data demonstrate that each deletion virus had similar but nonoverlapping host responses to infection in K18-hACE2 mice, which will aid in identifying the role of each infection.

## Discussion

A unique characteristic of coronaviruses is the inclusion of genes in the 3′ end of the genome that are unique to each member of the coronavirus family (21). These genes are called “accessory genes” because, while unique to each family member, they are not essential for replication (22). Many of these genes across the coronavirus family have been shown to alter host pathways including IFN signaling, cell cycle progression, and other assorted anti-viral responses to viral infection, with this interference affecting viral replication and pathogenesis (9, 10, 23, 24).

We designed two ways to analyze the functional consequences of the accessory genes in SARS-CoV-2 in vivo. First, we produced deletion viruses that deleted each accessory ORF from our infectious clone of SARS-CoV-2. Second, we produced mutant SARS-CoV-2 viruses that contained the spike of each previously circulating variant in the WA-1 background. As variants of SARS-CoV-2 have emerged, the increasing incidence of mutations both within spike and outside of spike were noted. Although the mutations in spike may enhance the entry and kinetics of infection, the mutations observed in the other genes may alter disease severity through interactions with the host immune system. In our experiments, we sought to differentiate between the role of spike mutations in each variant and those in other genes of the variant genome. As many of these nonspike mutations in the genome are in the accessory ORFs, we decided to couple our work with our variant spike in WA-1 viruses with our work with accessory deletion viruses. There are mutations in ORF1ab in the variants as well as the accessory ORFs; however, in this work we specifically investigated the role of accessory deletions. This allowed us to determine which accessory ORFs contribute significantly to pathogenesis through infection with our deletion viruses and to begin to link mutations in the corresponding variant ORFs to impacts on ORF function and pathogenesis.

Our work with the accessory deletion viruses has revealed that ORF3a and ORF3b contribute significantly to viral replication in K18-hACE2 mice. Mice infected with a WA-1ΔORF3a/b deletion virus demonstrated attenuated weight loss and significantly reduced lung titers at both day 2 and day 4. Our finding that an ORF3a/b deletion virus was attenuated in mice is supported by previously published work, as is our finding that WA-1ΔORF6 was not attenuated in mice (25). Silvas et al. also found that deletions of ORF7a, ORF7b, and ORF8 showed significantly reduced lung titer, which we did not find in our experiments, although we did find significant differences in lung inflammation in the WA-1ΔORF8 virus. This may be due to inoculation differences in our model, where mice were infected with a nonlethal dose of 10^3^ pfu instead of 10^5^ pfu as used in the previous study. It is possible that we may see attenuation in weight loss and viral replication of WA-1ΔORF7a/7b and WA-1ΔORF8 at higher doses or in different mouse backgrounds, which were not studied here.

The lung cytokine and chemokine profiles of mice infected with WA-1ΔORF3a/b demonstrated reduced expression of inflammatory cytokines and chemokines compared to WA-1. This difference is most likely attributable to the fact that the WA-1ΔORF3a/b virus demonstrated attenuated replication in mice. As expected, the attenuated replication of WA-1ΔORF3a/b resulted in the down-regulation of cytokines and chemokines involved in neutrophil recruitment, including Ccl7, Csf3, Cxcl1, Cxcl3, and Cxcl5. There was a small subset of genes that were up-regulated in WA-1ΔORF3a/b lungs. Two of these up-regulated genes are Il4 and Il5, which function to drive the T helper 2 (Th2) response. Interestingly, COVID-19 is known to skew to a Th2 response through stimulating the production of Il4 and Il5 (26). Given that the WA-1ΔORF3a/b virus is attenuated compared to WA-1, we would expect a down-regulation of these cytokines. The down-regulation of Il4 might in part be explained by its role in the tissue-remodeling process, with the faster clearance of WA-1ΔORF3a/3b allowing for tissue repair to take place (27). Another of these up-regulated genes, adipoq, encodes the insulin-sensitizing hormone adiponectin. Interestingly, reduced adiponectin levels are associated with severe respiratory failure in COVID-19 patients (28). Future work to characterize the role of these cytokines and chemokines in SARS-CoV-2 pathogenesis will focus on how these pathways interact with the accessory genes of the virus.

Although there were no statistically significant differences in lung titer, transcriptomic analysis revealed an unexpected difference in the lungs of mice infected with the WA-1ΔORF8 virus compared to WA-1 and the other deletions. Correlating with the histological data, where inflammation increased over the 4 d in the WA-1ΔORF8 deletion virus compared to WA-1, we saw an increased difference in whole-genome WA-1ΔORF8 responses in the mouse lungs. We also found a significant up-regulation of genes involved in wound healing that correlates with the histology data for that mutant. In addition, it was previously reported that ORF8 binds to the IL-17 receptor and acts as an agonist in the IL-17 signaling pathway (11). In our analysis, we did not see induction of the IL-17 pathway as previously reported. We hypothesized that without ORF8 to stimulate the pathway in vivo, we would see reduced induction of IL-17 signaling in these lungs. In our experiments, we did not see differences in induction of IL-17 or the associated pathway members at this dose. This could be a limitation of our bulk RNA sequencing approach, or it could be that the pathway at this timepoint at this dose is not strongly induced. Further evaluation of this pathway and the way that ORF8 interacts with it in vivo is warranted due to this discrepancy.

As the emergence of variants of SARS-CoV-2 occurred, we noted the appearance of point mutations in the accessory genes of SARS-CoV-2 as well as in the other structural and replicase genes. To elucidate the impact of nonspike mutations on viral pathogenesis, we synthesized variant spikes in the WA-1 backbone. We focused on the synthesis of the B.1.351, B.1.1.7, and P.1 lineages. When comparing the in vitro replication of the parent variants to their paired variant spikes in WA-1, we did not see many significant differences in replication in VeroE6 cells. The only significant difference we saw was a significant decrease in supernatant viral titer at 72 h of P.1 S in WA-1 compared to P.1.

In K18-hACE2 mice, the absence of in vitro replicative differences for B.1.351 S in WA-1 and B.1.1.7 in WA-1 was reflected. Mice infected with B.1.351 in WA-1 exhibited no differences in weight loss, lung titer, and brain titer compared to mice infected with B.1.351. This was also true for mice infected with B.1.1.7 compared to mice infected with B.1.1.7 S in WA-1. Results from the BALB/c experiment differed significantly for the B.1.351 S in WA-1 and B.1.351 pairing, with B.1.351 S in WA-1 showing attenuated weight loss and lung titers compared to B.1.351. The mice infected with B.1.1.7 S in WA-1 showed an increase in lung titer on day 2, but no difference in weight loss was seen compared to B.1.1.7.

The most significant replicative differences in both BALB/c and K18-hACE2 mice were seen with P.1 and P.1 S in WA-1. In both sets of mice, mice infected with P.1 S in WA-1 demonstrated increased weight loss and significantly higher lung titers by plaque assay and qPCR than mice infected with P.1. In K18-hACE2 mice, the brain RNA and brain titers trended with these data as well, although the values were not significant.

Given the significant differences in P.1 S in WA-1 and P.1 replication in vivo, we analyzed cytokine and chemokine gene expression on both day 2 and day 4 lungs of these mice. Compared to P.1, the P.1 S in WA-1 cytokines and chemokines profiled differed the most on day 2. Most of the significant changes seen involved the up-regulation of genes involved in neutrophil recruitment. The most up-regulated gene was cxcl5, which has been implicated as the major chemoattractant for neutrophils in SARS-CoV-2 and a major cause of inflammation (29). It is therefore not unexpected that this gene would be up-regulated due to the increased lung titer of P.1 S in WA-1 compared to P.1. The down-regulated genes of interest included thpok and Il5, which are important for driving the differentiation of CD4^+^ T cells. This finding may suggest that there is some inhibition of the CD4^+^ T cell recruitment pathway by the nonspike genes in WA-1 that is attenuated or absent in P.1. There is also evidence of the loss of early IFN antagonism in the nonspike genes in P.1, as IFN-γ levels in lungs from the P.1 S in WA-1 mice were reduced at day 2 compared to the P.1 mice, despite the higher lung titers in the mice infected with P.1 S in WA-1. However, this difference was not seen at day 4.

Together, these findings demonstrate that ORF3a/b and ORF8 have substantial roles in pathogenesis and host responses to SARS-CoV-2. It is interesting to note that two of the variant spikes in WA-1 viruses studied here, B.1.351 and P.1, possessed mutations in ORF3a, and all of the variants studied here possessed mutations in ORF8. We do not exclude the potential impact of other mutations in the variants, such as those seen in ORF1a/b and the other structural proteins, such as N, on viral pathogenesis. Coupled with the finding from our accessory deletion work that ORF3a/b and ORF8 contribute to pathogenesis, we suggest that the emergence of mutations in the variant accessory ORFs, particularly ORF3a and ORF8, contribute to the differences in viral pathogenesis seen in these variants compared to WA-1. Possible advantageous effects on viral fitness and likelihood of transmission of these mutations are supported by the continued identification of mutations in these ORFs in new variants. We interpret these data as suggesting that mutations outside of spike may be driving critical phenotypes of SARS-CoV-2 infection and disease. Although spike mutations may allow for better engagement of the receptor to facilitate entry into cells as well as evasion of antibodies, the mutations in the accessory proteins may be negatively impacting disease, leading to less severe clinical phenotypes (30). The balance of these two strategies may confer longer courses of virus replication and spread in the lungs while allowing for increased time for virus transmission from one infected person to their contacts. We hypothesize that this balance is critical for further evolution of SARS-CoV-2, and as more variants emerge, we will identify additional mutations outside of spike that contribute significantly to viral replication, transmission, and pathogenesis.

## Supplementary Material

Supplementary File

Supplementary File

## Data Availability

All study data are included in the article and/or supporting information.
