# Supplementary material for: SARS-CoV-2 variant spike and accessory gene mutations alter pathogenesis
Source: Proc Natl Acad Sci U S A. 2022 Aug 30;119(37):e2204717119. doi: 10.1073/pnas.2204717119 (PMC9477415; doi:10.1073/pnas.2204717119)
Supplement: Supplementary File [file pnas.2204717119.sapp.pdf]

Supplementary Figures

Table S1. Primer sequences for cloning SARS-CoV-2 genome fragments.

Primer name	Sequence	Purpose	SARS2-CoV-2 DNA Fragment
Con5UTRR	gttggttggtttgttacctgggaaggtataaacctttaattagggataacagggtaatcgggtaccgagctcgaattc	Vector primer	1a-1
ConHu1-6F	tggtgaagttatcacctttgacaatcttaagacacttcttattaccctgttatccctagatcctctagagtcgacctg		
5UTR-F	attaaaggtttataccttcccagg	DNA fragment primer	
1a(Hu1-6)-R	aagaagtgtcttaagattgtcaaag		
ConHu1-7R	tctaggtggaatgtggtaggattactagtgtaatatacactagggataacagggtaatcgggtaccgagctcgaattc	Vector primer	1a-2
ConHu1-11F	aatactttactattccttatgtcattcactgtactctgttattaccctgttatccctagatcctctagagtcgacctg		
1a(Hu1-7)-F	gtgtatattacactagtaatcctacc	DNA fragment primer	
1a(Hu1-11)-R	aacagagtacagtgaatgacataag		
ConHu1-12R	aaaggcaactacatgactgtattcaccaaaagctcttctatagggataacagggtaatcgggtaccgagctcgaattc	Vector primer	1a-3
Con1aF	gcacaggcactagtactgatgtcgtatacagggcttttgaattaccctgttatccctagatcctctagagtcgacctg		
1a(Hu1-12)-F	tagaagagcttttggtgaatacagtc	DNA fragment primer	
1a-R	tcaaaagccctgtatacgacatc		
Con1bR	cgcacggtgtaagacgggctgcacttacaccgcaaacccgtagggataacagggtaatcgggtaccgagctcgaattc	Vector primer	1b-1
ConHu1-19F	tgtgagaaggcattaaaatatttgcctatagataaatgtaattaccctgttatccctagatcctctagagtcgacctg		
1b-F	cgggtttgcggtgtaagtgc	DNA fragment primer	
1b(Hu1-19)-R	tacatttatctataggcaaatattttaatgc		
ConHu1-20R	tagtgcatcaacagcggcatgagagcaagctgtatacacttagggataacagggtaatcgggtaccgagctcgaattc	Vector primer	1b-2
Con1bF	cagagttgttatttctagtgatgttcttgttaacaactaaattaccctgttatccctagatcctctagagtcgacctg		
1b(Hu1-20)-F	agtgtatacagcttgctctcatg	DNA fragment primer	
1b-R	ttagttgttaacaagaacatcactag		
ConSR	ttgttttctctaattataagtctacctttactaagaagagtagggataacagggtaatcgggtaccgagctcgaattc	Vector primer	S
ConSF	aactgtaactttgaagcaaggtgaaatcaaggatgctactattaccctgttatccctagatcctctagagtcgacctg		
S-F	ctcttcttagtaaaggtagacttataa	DNA fragment primer	
S-R	agtagcatccttgatttcaccttg		
Con3endR	ccaattgtgaagattctcataaacaaatccataagttcgttagggataacagggtaatcgggtaccgagctcgaattc	Vector primer	AP
Con3endF	ctatccccatgtgattttaatagcttcttaggagaatgacattaccctgttatccctagatcctctagagtcgacctg		
3end-F	acgaacttatggatttgtttatgagaat	DNA fragment primer	
3UTR-R	gtcattctcctaagaagctattaaaatc		

Table S2. Detection primers to screen positive SARS-CoV-2 DNA fragment clones.

Primer name	Sequence	Junction	Amplicon size (bp)	DNA Fragment
RCO495	acgacggccagtgaattg	5'	251	1a-1
Det5UTRR	cgagttactcgtgtcctgtc			
Det1aIntHu1-6F	cttcacctgatgctgttacagc	3'	361	
RCO493	gtctcacctaaatagcttgg			
RCO495	acgacggccagtgaattg	5'	465	1a-2
Det1aIntHu1-7R	ccattaacttgtgggtatttccac			
Det1aIntHu1-11F	gattctgagtactgtaggcacg	3'	456	
RCO493	gtctcacctaaatagcttgg			
RCO495	acgacggccagtgaattg	5'	422	1a-3
Det1aIntHu1-12R	gtgcacagcgcagcttcttc			
Det1aF	gctgtagatgctgctaaagcttac	3'	579	
RCO493	gtctcacctaaatagcttgg			
RCO495	acgacggccagtgaattg	5'	600	1b-1
Det1bR	ggcgtacacgttcacctaag			
Det1bIntHu1-19F	gctgacatcacatacagtaatgc	3'	427	
RCO493	gtctcacctaaatagcttgg			
RCO495	acgacggccagtgaattg	5'	391	1b-2
Det1bIntHu1-20R	gtggtgcaggtaattgagcag			
Det1bF	ggtggacagcctttgttactaatg	3'	445	
RCO493	gtctcacctaaatagcttgg			
RCO495	acgacggccagtgaattg	5'	535	S
DetSR	gtagcgttattaacaataagtaggg			
DetSF	gtaataggaattgtcaacaacacag	3'	631	
RCO493	gtctcacctaaatagcttgg			
RCO495	acgacggccagtgaattg	5'	501	AP
Det3aR	gaacggcatttccagcaaagc			
Det3UTRF	cacgcggagtacgatcgag	3'	245	
RCO493	gtctcacctaaatagcttgg			

Table S3. Primer sequences to generate and confirm SARS-CoV-2 mutants.

Primer name	Sequence	Description
S-F	ctcttcttagtaaaggtagacttataa	Primers to generate B.1.351 spike gene fragments
SA_C21614T_R	gagttctggttgtaaaattaacacactgact	
SA_C21614T_F	agtcagtgtgttaattttacaaccagaactc	
SA_A21801C_R	ggtaggacagggttagcaaacctcttagtac	
SA_A21801C_F	gtactaagaggtttgctaaccctgtcctacc	
SA_A22206G_R	aaaccctgagggagaccacgcactaaattaa	
SA_A22206G_F	ttaatttagtgcgtggtctccctcagggttt	
SA_G22299T_R	ggagtcaaataacttatatgtaaagcaagta	
SA_G22299T_F	tacttgctttacatataagttatttgactcc	
SA_G22813T_R	attataatcagcaatatttccagtttgccct	
SA_G22813T_F	agggcaaactggaaatattgctgattataat	
SA_G23012A_R	aacaattaaaacctttaacaccattacaagg	
SA_G23012A_F	ccttgtaatggtgttaaaggttttaattgtt	
UK&SA_A23063T_R	ggtaaccaacaccataagtgggttggaaacc	
UK&SA_A23063T_F	ggtttccaacccacttatggtgttggttacc	
SA_A23403G_R	tctgtgcagttaacaccctgataaagaacag	
SA_A23403G_F	ctgttctttatcagggtgttaactgcacaga	
SA_C23664T_R	gcaactgaattttctacaccaagtgacatag	
SA_C23664T_F	ctatgtcacttggtgtagaaaattcagttgc	
S_3000_R	atttgcacttcagcctcaac	
S_3000_F	tcctttcacgtcttgacaaa	
S-R	agtagcatccttgatttcaccttg	
S-F	ctcttcttagtaaaggtagacttataa	Primers to generate B.1.1.7 spike gene fragments
UK_21765–21770del_R	ttggttccatgctatctctgggaccaatgg	
UK_21765–21770del_F	ccattggtcccagagatagcatggaaccaa	
UK_21991–21993del_R	ttgttgtttttgtggtaaacacccaaaaat	
UK_21991–21993del_F	atttttgggtgtttaccacaaaaacaacaa	
UK&SA_A23063T_R	ggtaaccaacaccataagtgggttggaaacc	
UK&SA_A23063T_F	ggtttccaacccacttatggtgttggttacc	
UK_C23271A_R	gcatcagtagtgtcatcaatgtctctgccaa	
UK_C23271A_F	ttggcagagacattgatgacactactgatgc	
SA_A23403G_R	tctgtgcagttaacaccctgataaagaacag	
SA_A23403G_F	ctgttctttatcagggtgttaactgcacaga	
UK_C23604A_R	ctacgtgcccgccgatgagaattagtctgag	
UK_C23604A_F	ctcagactaattctcatcggcgggcacgtag	

UK_C23709T_R	ctaatagtaaaatttatgggtatggcaatag	
UK_C23709T_F	ctattgccatacccataaattttactattag	
UK_T24506G_R	ctttgtcaagacgtgcaaggatatcatttaa	
UK_T24506G_F	ttaaatgatatccttgcacgtcttgacaaag	
UK_G24914C_R	acacaaatgtgttgtgtgtagtaatgatttg	
UK_G24914C_F	caaatcattactacacacaacacatttgtgt	
S-R	agtagcatccttgatttcaccttg	
S-F	ctcttcttagtaaaggtagacttataa	Primers to generate P.1 spike gene fragments
BR_21614-21638_R	aattagtgtatgcagagggtaattgagttctgtttgtaaaattaacacactgact	
BR_21614-21638_F	agtcagtgtgttaattttacaaacagaactcaattaccctctgcatacactaatt	
BR_G21974T_R	cacccaaaaatggataattacaaaattgaaa	
BR_G21974T_F	tttcaattttgtaattatccatttttgggtg	
BR_G22132T_R	cttaaacacaaattcactaagatttttgaaa	
BR_G22132T_F	tttcaaaaatcttagtgaatttgtgtttaag	
BR_A22812C_R	ttataatcagcaatcgttccagtttgccctg	
BR_A22812C_F	cagggcaaactggaacgattgctgattataa	
SA_G23012A_R	aacaattaaaacctttaacaccattacaagg	
SA_G23012A_F	ccttgtaatggtgttaaaggttttaattgtt	
UK&SA_A23063T_R	ggtaaccaacaccataagtgggttggaaacc	
UK&SA_A23063T_F	ggtttccaacccacttatggtgttggttacc	
SA_A23403G_R	tctgtgcagttaacaccctgataaagaacag	
SA_A23403G_F	ctgttctttatcagggtgttaactgcacaga	
BR_C23525T_R	atgagttgttgacatattcagcccctattaa	
BR_C23525T_F	ttaataggggctgaatatgtcaacaactcat	
BR_C24642T_R	cactctgacattttaatagcagcaagattag	
BR_C24642T_F	ctaatcttgctgctattaaaatgtcagagtg	
BR_G25088T_R	tttgaatgtttacaaatgaagcattaatgcc	
BR_G25088T_F	ggcattaatgcttcatttgtaaacattcaaa	
S-R	agtagcatccttgatttcaccttg	
CRISPRFCR1ORF3a	taatacgactcactatagggcgaggagctgttcaccggttttagagctagaaatagcaa	F- primers to generate CRISPR sgRNA for ΔORF3a
CRISPRFCR2ORF3a	taatacgactcactataggcgagagtgatcccggcgggttttagagctagaaatagcaa	
DelOligoS3a	tgctcaaaggagtcaaattacattacacataaacgaacttgcacaagctgatgagtacgaacttatgtactcattcgttt	DNA fix for ΔORF3a mutation
CRISPRFCR1ORF6	taatacgactcactataggtgtaatcaagattccaaagttttagagctagaaatagcaa	F- primers to generate CRISPR sgRNA for ΔORF6
CRISPRFCR2ORF6	taatacgactcactataggtagatgaagagcaaccaagttttagagctagaaatagcaa	
ORF6delfix	agtgacaatattgctttgcttgtacagtaagtgacaacagacgaacatgaaaattattcttttcttggcactgataacac	DNA fix for ΔORF6 mutation
CRISPRFCR1ORF7a	taatacgactcactataggctcttctggaacatacgagttttagagctagaaatagcaa	F- primers to generate CRISPR sgRNA for ΔORF7a
CRISPRFCR2ORF7a	taatacgactcactataggaaatttgttatcagctaggttttagagctagaaatagcaa	

DelOligo7a	attagatgaagagcaaccaatggagattgattaaacgaacttgaactttcattaattgacttctatttgtgctttttagc	DNA fix for ΔORF7a mutation
CRISPRFCR1ORF8	taatacgactcactataggcgatatcgatgtactgaagttttagagctagaaatagcaa	F- primers to generate CRISPR sgRNA for ΔORF8
CRISPRFCR2ORF8	taatacgactcactataggctttaattgaattgtgcggttttagagctagaaatagcaa	
ORF8delfix	actgcaagatcataatgaaacttgtcacgcctaaacgaacacgaacaaactaaaatgtctgataatggaccccaaaatca	DNA fix for ΔORF8 mutation
3aF	gtaataggaattgtcaacaacacag	Primers to confirm ORF gene deletions in AP fragment
3aR	GCCATAACAGCCAGAGGAAA	
6/7aF	TTGCTACATCACGAACGCTT	
6R	gttcgtttaggcgtgacaagtttc	
7aR	gaatgggtgatttagaaccagcc	
8F	ttgaactttcattaattgacttctatttgtg	
8R	ctgccttgtgtggtctgcatg	

Table S4. Primer sequences to construct SARS-CoV-2 complete genome vector.

Primer name	Sequence	Description
CMVpromF	attaccctgttatccctagttgacattgattattgactagttat	F and R primers to amplify CMV promoter from HCMV Toledo genome
CMVpromR	gttggttggtttgttacctgggaaggtataaacctttaatacggttcactaaacgagctctgcttatatagacc	
PA35-HDV fix	tggagatgccatgccgaccctttttttttttttttttttttttttttttttttttgtcattctcctaagaagcta	Fix with polyA sequence
Pcmv-BamHI fix	tataccttcccaggtaacaaaccaaccaacggatccctatccccatgtgattttaatagcttcttaggagaatgac	Fix to introduce BamHI site
Hu1-34	tatggaagagccctaatgtgtaaaattaattttagtagtgctatccccatgtgattttaatagcttcttaggagaatgacaaaa aaaaaaagggtcggcatggcatctccacctcctcgcggtccgacctgggcatccgaaggaggacgtcgtccactcgg atggctaagggagagctcggatcgatccgcctcgactgtgccttctagttgccagccatctgttgtttgcccctcccccgt gccttccttgaccctggaaggtgccactcccactgtcctttcctaataaaatgaggaaattgcatcgcattgtctgagtagg tgtcattctattctggggggtggggtggggcaggacagcaagggggaggattgggaagacaatagcaggcatgctg gggagagctcgagctctgtacatgtccgcgga	gBlock with HDV Rz and BGH terminator
ConCMVpR	attgattactattaataactagtcaataatcaatgtcaactagggataacagggtaatcgggtaccgagctcgaattc	pCC1-Bac-YCp-URA vector construction primers
ConBGHtermF	gcatgctggggagagctcgagctctgtacatgtccgcggaattaccctgttatccctagatcctctagagtcgacctg	

Table S5. Detection primers to screen positive SARS-CoV-2 complete genomes.

Primer name	Sequence	Junction
RCO495	acgacggccagtgaattg	J1
DetCMVpR	ggtcatgtactgggcataatgc	
DetCMVpF	gcattatgcccagtacatgacc	J2
Det5UTRR	cgagttactcgtgtcctgtc	
Det1aIntHu1-6F	cttcacctgatgctgttacagc	J3
Det1aIntHu1-7R	ccattaacttgtgggtatttccac	
Det1aIntHu1-11F	gattctgagtactgtaggcacg	J4
Det1aIntHu1-12R	gtgcacagcgcagcttcttc	
Det1aF	gctgtagatgctgctaaagcttac	J5
Det1bR	ggcgtacacgttcacctaag	
Det1bIntHu1-19F	gctgacatcacatacagtaatgc	J6
Det1bIntHu1-20R	gtggtgcaggtaattgagcag	
Det1bF	ggtggacagcctttgttactaatg	J7
DetSR	gtagcgttattaacaataagtaggg	
DetSF	gtaataggaattgtcaacaacacag	J8
Det3aR	gaacggcatttccagcaaagc	
Det3UTRF	cacgcggagtacgatcgag	J9
DetBGHtermR	gaaaggacagtgggagtggc	
DetBGHtermF	gccactcccactgtcctttc	J10
RCO493	gtctcacctaaatagcttgg	

Table S6. Variant spike mutations**.**

Lineage	Variant spike amino acid substitutions vs WA1
B.1.351	L18F, D80A, D215G, R246I, K417N, E484K, N501Y, D614G, A701V
B.1.1.7	HV 69–70del, Y144del, N501Y, A570D, D614G, P681H, T716I, S982A, D1118H
P.1	L18F, T20N, P26S, D138Y, R190S, K417T, E484K, N501Y, D614G, H655Y, T1027I, V1176F

Table S7. Fold changes of inflammatory cytokines and chemokines of mouse lungs on Day 2 and Day 4.

**Experimental Group**	**WA-1**	**WA-1ΔORF3a/b**	**P.1 S in WA-1**
**Control Group**	**PBS**	**WA-1**	**P.1**

	**Fold Change Relative to Control Group**					
**Gene**	**Day 2**	**Day 4**	**Day 2**	**Day 4**	**Day 2**	**Day 4**
Adipoq	**0.32**	**4.78**	**9.64**	**9.23**	**0.36**	**2.00**
Bmp2	0.94	**0.44**	0.70	0.86	1.12	0.87
Bmp4	**0.45**	**0.28**	1.10	1.37	0.64	0.94
Bmp6	**0.41**	**0.21**	1.71	0.87	0.69	0.77
Bmp7	**0.19**	**0.33**	**2.23**	1.32	0.57	0.69
Ccl1	1.84	**6.92**	**0.45**	1.04	**2.91**	1.27
Ccl11	**2.48**	1.92	1.15	1.11	1.14	0.55
Ccl12	**29.84**	**10.44**	**0.37**	0.70	1.59	0.95
Ccl17	0.85	**0.37**	0.93	**3.13**	0.51	0.93
Ccl19	1.55	1.04	0.76	0.93	1.10	0.99
Ccl2	**235.79**	**68.76**	**0.17**	0.77	**2.66**	0.63
Ccl20	1.94	**10.45**	**0.31**	0.92	**2.71**	1.64
Ccl22	**2.10**	0.61	1.11	1.91	0.94	0.81
Ccl24	1.16	0.54	**0.46**	1.06	0.66	1.23
Ccl3	**9.93**	**4.51**	**0.29**	**0.34**	1.60	0.78
Ccl4	**25.27**	**7.00**	**0.21**	**0.27**	1.57	0.62
Ccl5	**3.09**	1.82	0.56	0.64	1.66	1.03
Ccl7	**207.07**	**72.81**	**0.16**	0.73	**2.47**	0.70
Cd40lg	0.51	0.55	1.05	0.82	0.94	0.93
Cd70	0.89	1.11	**0.48**	1.64	1.53	0.69
Cntf	0.76	0.74	0.87	1.15	0.58	0.93
Csf1	**3.02**	1.53	1.33	0.71	1.42	0.64
Csf2	1.58	1.19	0.90	0.90	**0.41**	0.88
Csf3	**7.03**	**7.07**	**0.08**	**0.26**	**3.49**	0.86
Ctf1	**0.33**	0.56	**2.38**	1.09	0.74	0.96
Cx3cl1	0.69	**0.40**	1.25	0.72	1.18	0.66
Cxcl1	**8.57**	**3.55**	**0.13**	1.53	**2.47**	0.71
Cxcl10	**505.97**	**84.16**	**0.49**	**0.49**	1.67	**0.40**
Cxcl11	**12.04**	**3.85**	**2.11**	1.71	1.75	0.59
Cxcl12	**0.44**	0.77	1.62	0.96	0.85	1.37
Cxcl13	**5.36**	**7.55**	0.83	0.83	1.42	1.63
Cxcl16	**2.65**	**2.12**	1.00	1.31	1.68	0.97
Cxcl3	**32.32**	**7.27**	**0.09**	1.03	1.37	0.94

Cxcl5	**6.97**	**5.15**	**0.06**	0.80	**4.23**	1.17
Cxcl9	**35.48**	**9.00**	**0.36**	1.77	1.65	0.78
Fasl	**2.21**	1.92	0.59	0.94	1.22	1.04
Gpi1	0.59	**0.48**	1.13	1.12	0.72	1.09
Hc	0.54	**0.27**	1.14	1.39	0.65	0.65
Ifna2	**6.48**	1.05	0.52	**0.46**	1.29	**0.17**
Ifng	**16.38**	**2.34**	**0.46**	0.63	1.01	**0.41**
Il10	**7.00**	**7.16**	**0.45**	**0.27**	1.63	0.78
Il11	1.89	1.59	**0.48**	1.19	1.18	1.47
Il12a	**0.27**	**0.30**	1.73	0.98	**0.35**	0.85
Il12b	1.99	**4.18**	0.76	**0.34**	0.94	1.04
Il13	1.21	0.61	0.66	1.44	0.82	1.24
Il15	**2.87**	0.83	0.75	0.74	0.93	0.69
Il16	0.57	0.59	1.45	0.99	1.09	0.94
Il17a	1.33	0.54	**0.40**	0.72	0.66	0.95
Il17f	1.12	0.73	0.86	1.03	1.58	0.78
Il18	1.30	0.63	0.61	1.10	1.09	0.84
Il1a	1.58	**0.49**	**0.48**	1.38	0.90	0.77
Il1b	**2.22**	**0.43**	**0.23**	0.90	**2.02**	0.74
Il1rn	**22.45**	**6.31**	**0.23**	**0.45**	**2.18**	**0.50**
Il2	1.22	**0.45**	**0.45**	0.80	0.70	0.95
Il21	1.22	0.54	**0.44**	0.72	**0.46**	0.95
Il22	1.22	0.54	**0.44**	0.72	0.66	0.95
Il23a	**4.07**	**2.10**	**0.34**	0.91	**2.65**	0.76
Il24	1.27	**5.67**	**0.37**	0.97	**2.15**	0.95
Il27	**3.45**	1.30	0.56	0.54	1.29	0.90
Il3	1.22	0.54	**0.44**	0.72	0.66	0.95
Il4	**0.25**	**0.19**	**2.13**	1.33	0.64	1.05
Il5	**0.30**	**0.30**	**2.23**	0.72	**0.48**	0.75
Il6	**58.13**	**14.27**	**0.27**	1.02	**2.47**	**0.37**
Il7	0.65	0.55	1.08	1.55	0.79	0.94
Il9	1.22	0.54	**0.44**	0.72	0.66	0.95
Lif	1.72	**4.76**	1.48	1.48	1.30	1.53
Lta	**2.49**	1.59	1.05	**0.41**	0.59	0.62
Ltb	0.57	0.85	1.41	0.86	0.81	0.81

Mif	0.84	**0.44**	0.71	1.40	0.76	0.96
Mstn	1.08	0.54	0.53	1.27	**0.17**	0.75
Nodal	1.22	**0.27**	**0.44**	0.72	0.59	0.97
Osm	**2.60**	1.00	**0.31**	0.85	**2.72**	0.76
Pf4	**0.49**	**0.24**	1.50	0.74	0.60	1.10
Ppbp	0.63	**0.19**	1.83	**0.50**	0.59	0.87
Spp1	**0.39**	1.49	1.01	1.02	1.38	1.55
Tgfb2	0.52	**0.27**	0.85	1.00	0.76	0.82
Thpo	0.83	0.73	0.80	0.88	**0.46**	1.60
Tnf	**13.22**	**4.57**	**0.31**	**0.31**	1.33	0.86
Tnfrsf11b	1.18	0.78	0.83	0.94	1.08	0.98
Tnfsf10	1.15	**0.41**	0.98	0.77	0.81	0.60
Tnfsf11	**0.30**	0.78	1.29	1.83	**0.36**	1.16
Tnfsf13b	1.02	0.94	1.05	0.85	0.90	0.86
Vegfa	**0.49**	**0.25**	1.41	0.90	0.62	0.59
Xcl1	**6.85**	**6.98**	0.52	0.67	1.40	0.66
Actb	1.03	0.77	0.90	0.92	1.09	0.88
B2m	**2.32**	**2.07**	0.82	1.28	0.93	1.01
Gapdh	0.94	1.03	1.22	1.32	0.87	0.86
Gusb	0.59	1.09	1.34	0.74	0.91	1.17
Hsp90ab1	0.74	0.56	0.83	0.86	1.25	1.11

Table S8. Excel file with RNAseq fold change values for knockout viruses; all genes (tab a) and only ISGs (tab b).

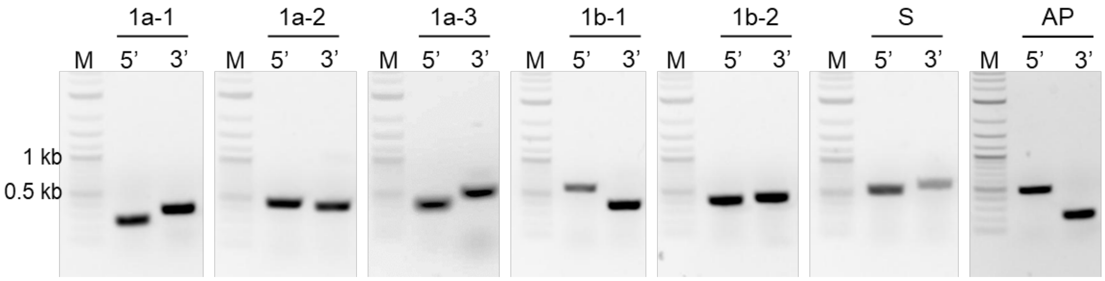

Figure S1. PCR confirmation of SARS-CoV-2 genome fragments used to assemble full-length genomes. Junctions between vector and each SARS-CoV-2 DNA fragment were PCR amplified using detection primers in Table S2. 5', junction at 5' end of SARS-CoV-2 fragment; 3', junction at 3' end of SARS-CoV-2 fragment; M: 2-log marker.

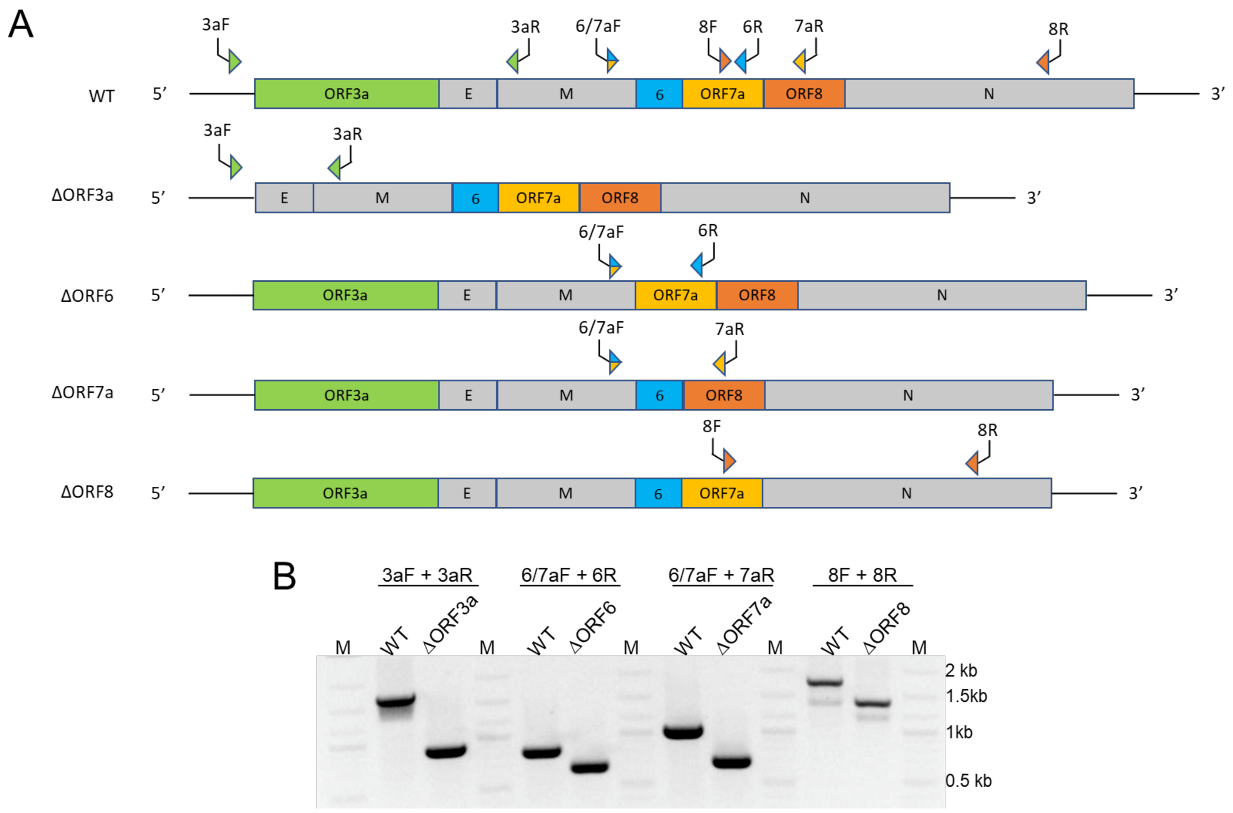

Figure S2. PCR amplification to confirm accessory ORF deletions. A. Schematic of SARS-CoV-2 WA1 accessory ORF deletion mutants (ΔORF3a, ΔORF6, ΔORF7a, ΔORF8). B. PCR screen to test for ORF deletions in mutants. Primer sequences are listed in Table S3. WT, wild type WA1; M: 2-log marker.
